# Role of ARP2/3 Complex-Driven Actin Polymerization in RSV Infection

**DOI:** 10.3390/pathogens11010026

**Published:** 2021-12-26

**Authors:** Autumn Paluck, Jaspreet Osan, Lauren Hollingsworth, Sattya Narayan Talukdar, Ali Al Saegh, Masfique Mehedi

**Affiliations:** 1 School of Medicine and Health Sciences, University of North Dakota, Grand Forks, ND 58202, USA; autumn.paluck@und.edu (A.P.); jko4001@med.cornell.edu (J.O.); lauren.buchholtz@und.edu (L.H.); sattyanarayan.talukd@und.edu (S.N.T.); ali.alsaegh@und.edu (A.A.S.); 2 Department of Radiation Oncology, Weill Cornell Medicine, New York, NY 10065, USA

**Keywords:** cytoskeleton dynamics, filopodia, ARP2/3 complex, actin polymerization, cell-to-cell spread, RSV, bronchiolitis, therapeutics

## Abstract

Respiratory syncytial virus (RSV) is the leading viral agent causing bronchiolitis and pneumonia in children under five years old worldwide. The RSV infection cycle starts with macropinocytosis-based entry into the host airway epithelial cell membrane, followed by virus transcription, replication, assembly, budding, and spread. It is not surprising that the host actin cytoskeleton contributes to different stages of the RSV replication cycle. RSV modulates actin-related protein 2/3 (ARP2/3) complex-driven actin polymerization for a robust filopodia induction on the infected lung epithelial A549 cells, which contributes to the virus’s budding, and cell-to-cell spread. Thus, a comprehensive understanding of RSV-induced cytoskeletal modulation and its role in lung pathobiology may identify novel intervention strategies. This review will focus on the role of the ARP2/3 complex in RSV’s pathogenesis and possible therapeutic targets to the ARP2/3 complex for RSV.

## 1. Introduction

Microorganisms, including viruses, use the host cell’s cytoskeleton to destabilize the host cell’s physiological mechanisms to allow for the virus’s survival and aid its pathogenesis. Most bacteria and viruses utilize the host cytoskeleton for multiple activities, including attachment, invasion, movement within and between cells, and replication, resulting in disease progression [1,2,3]. Actin microfilaments are unique among the cellular cytoskeletons, as they are composed of a highly dynamic network of actin polymers. Host cells contain actin-associated proteins that modulate cell migration, contraction, and shape changes during the cell cycle and in response to extracellular stimuli [4,5,6]. During a microbial attack, the induction of macropinocytosis, phagocytosis, membrane ruffling, vacuole formation, and vacuole remodeling depends on signaling the actin cytoskeleton [4,5,6]. Pathogens, like viruses and bacteria, have different strategies to hijack the host cell machinery to promote their replicative cycles; specifically, the host cytoskeleton is their common target [3,4].

**Figure 1:**
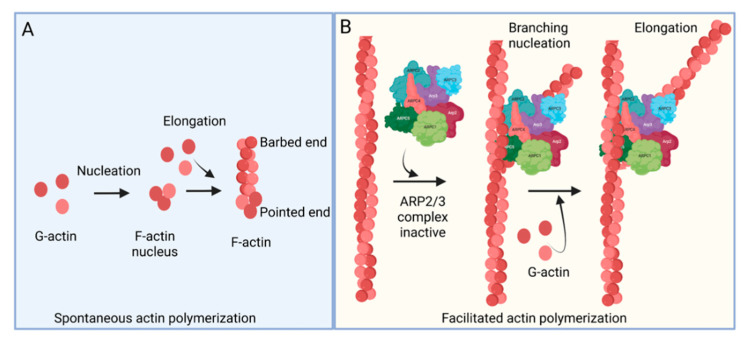
Actin polymerization. (**A**) Spontaneous actin polymerization. Globular actins (G-actin) form follicular actin (F-actin) nucleus (shown as pointed end). Spontaneous addition of G-actin elongates F-actin at the barbed end. (**B**) Facilitated actin polymerization. ARP2/3 complex, a seven-protein complex, consists of ARP2, ARP3, ARPC1, ARPC2, ARPC3, ARPC4, and ARPC5. ARP2/3 complex involves branched actin polymerization [22].

The ARP2/3 complex, an actin filament nucleating and regulating factor, plays a central role in cellular actin assembly. The complex is an assembly of seven proteins, including actin-related proteins ARP2, ARP3, and five additional subunits called actin-related protein 2/3 complex (ARPC), including p41, p34, p21, p20, and p16 (noted as ARPC 1–5, respectively) (Figure 1) [7]. Importantly, it regulates the actin cytoskeleton by nucleating branched actin filament networks, which are usually active at the leading edge of cells. The growth of these filaments produces a force that is able to protrude the membrane [8,9,10]. The ARP2/3 complex, localized in the lamellipodia, is composed of a heterodimer, consisting of ARP2 and ARP3, that is able to interact with the pointed (less dynamic) end of an actin subunit, but not the barbed (more dynamic) end. This leads to nucleated actin filament growth towards the barbed end (Figure 1) [11,12,13]. ARP2 mediated actin nucleation demonstrates in vitro branching and crosslinking of the actin filaments, but in vivo is thought to drive the formation of lamellipodia and act as a control center for actin-based motility [14,15,16]. The ARP2/3 complex plays an important role in lamellipodia extension and filopodia formation in spreading cells [17,18,19]. Filopodia are considered cell sensors because of their protrusive, retractile, and sweeping motility properties. Many cells use these features of filopodia to explore their environment and to form new adhesion contacts for motility and spreading [19,20]. It is proposed that filopodia arise from lamellipodia in response to additional signals and that the reorganization of the ARP2/3 complex is involved in this process [21].

**Figure 2:**
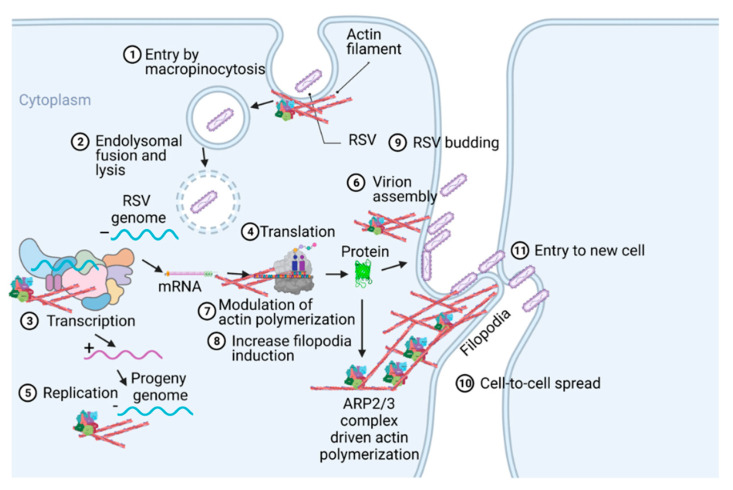
A pictogram of a replicative cycle of RSV. Different steps of RSV replicative cycle (including potential actin involvement) are indicated chronologically.

**Figure 3:**
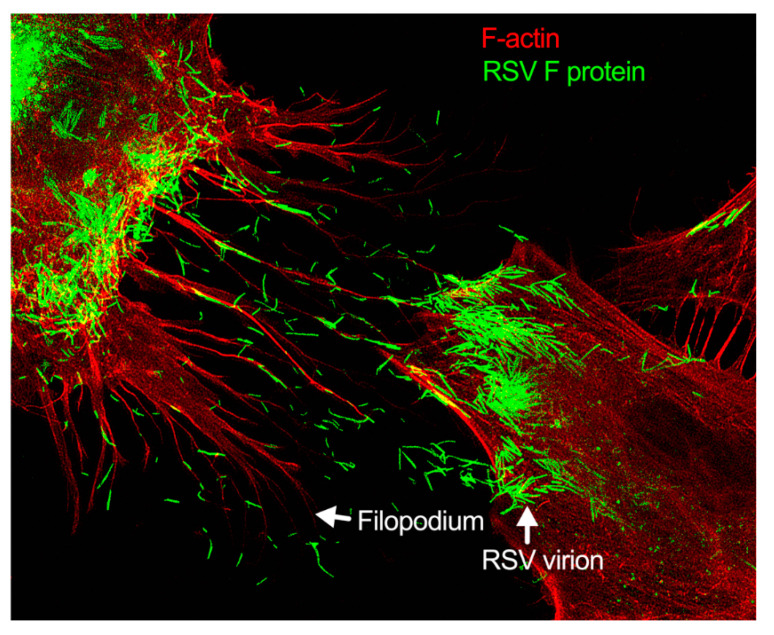
Filopodia-driven RSV cell-to-cell spread. Human lung airway epithelial cell line, A549 cells, were infected with RSV wild type (RSV-WT) (Strain A) at a multiplicity of infection (MOI) of 1 for 24 h. The infected cells were then fixed, permeabilized, and stained for RSV fusion (F) protein using antibody specific to F. F-actin and the nucleus were stained with rhodamine phalloidin and DAPI, respectively. The image was taken under a stimulated emission depletion (STED) microscope (Leica Microsystem) [41,42].

Respiratory syncytial virus (RSV) causes severe lower respiratory illnesses, such as bronchiolitis and pneumonia, in children under the age of five. Elderly adults, as well as adults with chronic diseases, are at an increased risk for contracting a severe illness from an RSV infection. Importantly, almost everyone has been infected by RSV by the time they reach two years of age. RSV infection primarily causes common cold-like symptoms that progress to lower respiratory tract disease in 40 percent of infected infants [23]. RSV belongs to the *Pneumoviridae* virus family. It is a negative-sense, single-stranded, non-segmented RNA virus. Its genome consists of 10 genes that encode 11 proteins. The proteins encoded by RSV are nonstructural protein 1(NS1), NS2, nucleoprotein (N), phosphoprotein (P), matrix (M), short hydrophobic (SH), glycoprotein (G), fusion (F), M2-1 and M2-2, and RNA-dependent RNA polymerase (L) [24]. The two main proteins that are widely studied for anti-viral drug discovery are F and G protein. F protein is a surface glycoprotein that is involved in the RSV infection. G protein attaches to the host cell receptor [25]. F protein enables the virion membrane to fuse with the host cell membrane [26]. Upon entering the host cell, RSV undergoes transcription, translation, and replication in the host cytoplasm [24]. During transcription, the viral polymerase starts mRNA synthesis for all genes from 3′ to 5′ end of the genome [27]. Importantly, RSV 3′ to 5′ end genes undergo a higher to lower transcription gradient [27,28]. All protein-specific mRNAs are translated by host cell translational machinery [24]. N protein is involved in creating a template for RNA synthesis and P protein is a polymerase co-factor [24]. M protein is involved in the inhibition of host transcription and is associated with viral inclusion bodies [29]. M2-1 is a transcription processivity factor and M2-2 regulates RNA synthesis [30,31]. NS1 and NS2 proteins are non-structural proteins that are involved in various processes including interfering with the innate immune response and inhibiting apoptosis [32,33]. SH protein is a transmembrane protein near the N-terminus involved in various processes including the inhibition of TNF signaling and reducing apoptosis [34]. The virus is assembled at the cell surface with viral proteins and genomic RNA. The assembled new virion budded out from the surface of the cell [24]. RSV attachment to cell membrane activated various signaling cascades like Epidermal growth factor (EGF), cell division protein 42 (Cdc42) which led to actin rearrangement and increased fluid uptake which results in RSV uptake by macropinocytosis, as shown in Figure 2. Actin rearrangement plays an important role in RSV entry, as treatment with cytochalasin D and latrunculin A disrupt actin filament and reduce RSV infection in HeLa cells [35]. Previously, it has also shown that the cytoskeleton protein actin is involved in RSV endocytosis, replication, gene expression, and morphogenesis (Figure 2) [36,37,38,39]. It has recently been shown that ARP2/3 and virus-induced filopodia contribute to RSV cell-to-cell spread (Figure 2 and Figure 3) [40,41,42].

## 2. Pathogen-Induced Subversion of Cytoskeleton

The actin cytoskeleton is critically important to maintaining cellular morphology and motility. Pathogens co-opt the actin restructuring machinery of the host cell to access or create a favorable environment for their replication. Although the focus of this review is on RSV, the subversion of the host cytoskeleton is not limited to viral pathogens. Viruses are known to stimulate a rearrangement of the host cell actin cytoskeleton during the infection of the cell [43]. The actin cytoskeleton can control endocytosis and phagocytosis, as well as cell contraction, motility, and division [44]. The cytoskeleton’s capacity to continuously assemble and disassemble allows it to take on many roles [43]. Pathogens manipulate the cytoskeleton to drive cellular infection due to its role of being a major host structural component [45]. A pathogen will utilize effector proteins to hijack the cytoskeleton to successfully infect and replicate [45]. For example, actin reorganization is stimulated during microbial entry by binding with CR3 receptors in macrophage [46]. In contrast, translocated actin recruiting phosphoprotein (TARP) in non-phagocytic cell facilitates entry by promoting actin nucleation [47]. Cortactin, which is an actin regulatory protein, is also important for the entry in non-phagocytic cells [48]. ARP2/3 complex regulation by Src and PI-3 kinase were shown in bacterial internalization [49]. Actin-mediated propulsion is also necessary for cell-to-cell spread but not always dependent on N-WASP and ARP2/3 complex [50]. The ARP2/3 complex, as well as pathogen-induced filopodia, are used as mechanisms to subvert the cytoskeleton by stimulating actin assembly in the host cell [51]. The mechanism of the ARP2/3 complex is utilized in the formation of branched actin, while the activation of Cdc42 plays a role in the development and formation of filopodium [52]. Thus, various pathogens exploit the host cell actin cytoskeleton by implementing mechanisms to subvert it [51].

In contrast to some pathogens, including bacteria and fungi, viruses generally do not follow a symbiotic relationship. Viruses are dependent on the host transport system to traverse the cytoplasm [53]. Because of this, viruses have developed strategies that allow them to manipulate the normal functions of the cytoskeleton [53]. Viruses can interact with the actin cytoskeleton to initiate, sustain, and spread infection [38]. The promotion of the viral protein’s interaction with the actin cytoskeleton begins to redirect its structure and function [38]. The host cytoskeletal transport networks are a target of viruses, and, over time, the pathogens have developed ways to hijack the network to assist in their regulation and the facilitation of their movement [53]. There are various examples of virus modulating host cytoskeleton for viral infection. Vaccinia virus use microtubules to move virus to various phases of the replication cycle from forming intracellular mature virus to cell associated virions [54]. Influenza virus enters the cells via clathrin-dependent or independent pathway depending on the polarity of the cells [55,56]. It is also shown that actinin–4, a well-conserved protein which connects actin cytoskeleton to membrane and regulates cell motility, plays a role in Influenza A nuclear localization [57]. It has been shown that SARS-CoV N protein induces actin reorganization in monkey kidney cells [58]. Various members of paramyxovirus family use cytoskeleton protein for replication and assembly. Sendai virus and human parainfluenza virus use actin and tubulin for transcription and microfilaments for virus assembly [59,60].

## 3. Role of Cytoskeleton in RSV Infection Cycle

The host cytoskeleton is involved in various stages of the RSV infectious cycle (Table 1 and Figure 2 and Figure 3). RSV interacts with multiple cellular and cytoskeletal proteins during infection. Nucleolin located in the apical cell surface interacts with RSV glycoproteins which is important for RSV entry [61]. The cytoskeletal proteins, e.g., actin and microtubules, are implicated in the RSV transcription, assembly, and budding [62]. F protein cytoplasmic tail and M protein are responsible for viral assembly, specifically, F protein interacts with inclusion bodies which in turn facilitate the release of M protein-ribonucleoprotein complex from inclusion bodies [63]. RSV M protein interacts with actin which is important for budding and responsible for the virion particle transport [64]. Cytochalasin D inhibits actin polymerization, which reduces the production of viral particles [37]. RSV also increases the polymerization of actin, which results in cytoplasmic extension in the infected cells [36]. Profilin, an actin modulatory protein, is also essential for RSV transcription [65]. Apart from actin and profilin, actin-associated signaling pathways like RhoA, PI3K, and Rac GTPase are involved in the production of virus filaments [66,67,68]. Microtubules play a role in both assembly and release of the virus but have a greater impact on the assembly [39]. Higher expression of RhoA and pMLC2 were observed during RSV infection which induced stress fiber formation and ROCK inhibitor Y-27632 showed the inhibition of RhoA activity [69].

Generally, respiratory viruses enter the human via the lung epithelia. This can prove challenging, however, as most of the lung epithelia are lined with a protective coating of mucus. The few cells that are not lined with mucus are guarded by macrophages. This has made particle release, the common method of viral spread, disadvantageous for these types of viruses. To avoid these pitfalls, respiratory viruses have most likely evolved novel mechanisms of spreading cell-to-cell. RSV modulates cytoskeletal rearrangement and actin remodeling for its replicative cycle in vitro [39], particularly ARP2/3 complex-regulated filopodia formation for its cell-to-cell spread [40,41]. When A549 cells are infected with RSV, the cell forms an actin-based projection called filopodia (Figure 2 and Figure 3) [40,41]. Filopodia are finger-like projections comprised of polymerized actin. The free barbed ends of the actin filament can add additional actin monomers, which leads to actin polymerization [75]. The formation of the filopodia is activated by Cdc42, a protein involved in the regulation of proliferation in the cell division cycle [76,77,78,79].

## 4. Role of Filopodia in RSV Cell-to-Cell Spread

Filopodia are typically involved in activities such as migration and wound healing. Sites of filopodia formation tend to have increased amounts of the protein katanin [80]. Katanin, in these increased amounts, has been linked to more aggressive migratory behaviors in prostate cancer cells [81]. Cells using directional migration also use their filopodia to establish a single polarity axis. The cell’s filopodium serve as the leading process for directional migration [82,83,84]. Filopodia can form multiple protrusions branching out from the filopodia (or the lamellipodia), and these branches are often able to be used by the cell to make directional decisions in chemotaxis and pathfinding [85,86]. However, herein lies the difference between the cell’s filopodium and the cell’s lamellipodium: the role of the lamellipodium tends to be more reserved for migratory purposes. The thin sheet protrusion pulls the cell forward. In contrast, filopodia play a role that is less focused on pulling the cell forward but more so on exploring the extracellular environment. The filopodia are less sheet-like and more finger-like [87,88]. In certain cells, filopodia may also play a role in phagocytosis. For example, in certain non-polarized cell types, such as primary human corneal fibroblasts, or a cancerous cell line Chinese hamster ovary (CHO) cells stably expressing nectin-1, HSV-1 entry occurs through a mechanism that seems to be “phagocytosis-like”. It includes filopodia-like actin rearrangements and RhoA GTPase activation [89]. Conversely, Clement et al. also showed that two actin-depolymerizing agents—CytoD and latrunculin B (LatB)—are potent inhibitors of this HSV-1 entry in these cell types. Further studies in polarized retinal pigment epithelial (RPE) cells, which have a natural tropism for HSV-1 infection, also revealed the role of actin in facilitating virus entry [89].

The formation of filopodia in RSV-infected cells suggests filopodia may play a significant role in the spread of the respiratory syncytial virus (Figure 2 and Figure 3) [41]. The molecular link of Cdc42, N-WASP, and the ARP2/3 complex has a regulatory role in filopodia formation and actin polymerization [90]. Small GTP-binding proteins including Rac, Rho, and Cdc42 have distinct roles for actin cytoskeletal branching and regulation [91,92]. As an essential GTPase, Cdc42 is involved in multiple actin-dependent morphogenetic events by interacting with a myriad of downstream effectors [93]. N-WASP is a ubiquitously expressed protein and a member of the WASP family stimulated by upstream regulators Cdc42 and PI(4,5)P_2_ which transmit signals to the nucleation of actin filaments by the ARP2/3 complex [94,95]. The interaction between amino (NH_2_)-termini and the carboxy (COOH)-termini of N-WASP, having a regulatory role, can inhibit the actin nucleation activity. Additionally, an upstream regulator Cdc42 can activate N-WASP by binding, which results in lower affinity between these termini of N-WASP molecule [96,97,98].

During viral infection, viral proteins increase cell migration by disrupting and modulating actin dynamics to form long cellular extensions, like filopodia, and tunneling nanotubes in in vitro models [38]. It has already been shown that the ARP2/3 complex plays a significant role in some viral intracellular migration by altering actin polymerization [99,100]. In addition to the ARP2/3 complex, the Rho GTPases (RhoA and Cdc42) have been shown to involve the RSV replicative cycle. For example, the interaction between RhoA with the fusion glycoprotein (RSV-F) of RSV facilitates virus-induced syncytium formation [101]. Moreover, the ARP2/3 complex-depleted cells have shown increased RhoA activity which indicates that the ARP2/3 complex may affect cell motility by altering Rho GTPase signaling [17]. Whether any RSV proteins directly contribute to ARP2/3 complex actin polymerization and/or filopodia induction remains to be determined. However, a recent study has shown that F protein expression by using a viral vector induces filopodia [41].

## 5. RSV-Induced Modulation of Actin Signaling in Disease Pathophysiology

The actin cytoskeleton is essential for many immune cell functions, including migration, phagocytosis, activation, secretion, and cell–cell interaction, all of which are dependent on cytoskeletal mobilization. Thus, the actin cytoskeleton plays an important role in the host’s immune response to microbial infection. For example, the actin cytoskeleton directly involves the host’s innate immune response against *Salmonella* infection [102]. Alterations in several actin regulatory proteins have resulted in immune deficiency, autoimmunity, and autoinflammatory disease [103]. Importantly, altered cytoskeletal regulation is associated with immune pathology [103]. RSV infection can manifest mild upper respiratory tract illnesses and severe lower respiratory tract infections [104]. The histopathology of RSV infection in infants without treatment showed infection in the bronchial and alveolar epithelium and obstructed airways due to edema and inflammatory debris. The small airway obstruction could also be due to compression by lymphoid aggregates in the bronchiolar-associated lymphoid tissue (BALT) [105]. The cells infected with RSV included ciliated cells [106]. In the airway epithelium, immune cells like monocytes and lymphocytes, along with macrophages, were observed. The presence of this cell suggests that polymorphonuclear leukocytes play a role in RSV infection [107]. There is a difference between mice and human RSV infection when it comes to inflammation patterns. The inflammation is centered on the arteriole in the case of mice, while in humans the inflammation is more towards the bronchiole [105]. The immune system controls RSV infection by generating neutralizing antibodies and by the TH1-mediated immune response combined with inflammatory cells [108,109]. Despite the importance of reported RSV-induced cytoskeletal changes, there is no comprehensive information on how the virus-induced actin polymerization contributes to lung pathobiology. It is already known that cytoskeletal regulation plays a critical role in lung inflammation, as well as epithelial barrier function [110] and vascular endothelial barrier function [111]. Actin regulation is also involved in lymphocyte activation [112] and cytokine regulation [113]. Thus, we can infer that RSV-induced actin cytoskeleton modulation can play important roles in lung disease pathobiology, particularly in bronchial wall thickening (bronchiolitis).

## 6. Potential of RSV Therapeutics Targeting Localized Actin Dynamics

Currently, various drugs are being studied for the treatment of RSV. Ribavirin is the only approved drug for RSV infection, but due to its low efficacy, it is not routinely used [114]. Palivizumab, a prophylactic, is a monoclonal antibody targeted against the conserved epitope of F protein of RSV. It is administered to infants born prematurely or have cardiopulmonary disorders to prevent severe RSV infections [115].

When considering host actin as a therapeutic target of RSV infection, it is important to examine if this mechanism has been utilized in the past. For years, cell biologists have found mechanisms to influence the host actin cytoskeleton by using small-molecule modulators of actin [38]. Researchers have also found data that prove the cytoskeleton could be targeted to prompt antiviral protection due to the identification of a layer of host resistance [116]. When considering viral infections, the current research shows that virally induced diseases could be treated by targeting the actin cytoskeleton [38]. The current research on the host actin cytoskeleton, in terms of viral infections, warrants research exploring how the host actin cytoskeleton could be a target as a therapy for RSV infection. While numerous small molecules have been discovered that mimic the host regulatory mechanism for modulating the actin cytoskeleton, some of the compounds inhibit the ARP2/3 complex, both directly and indirectly, by inhibiting nuclear-promoting factors (NPFs), WASP, and WAVEs [117]. CK-666, a potent inhibitor of the ARP2/3 complex that blocks an activating conformational change [118], inhibits lung myofibroblast in vitro [119]. Additionally, synthetic triterpenoids, such as 2-cyano-3, 12-dioxooleana-1,9-dien-28-oic acid (CDDO)-IM, and CDDO-ME, target the ARP2/3 complex-driven branched actin polymerization and inhibit cell migration [120]. The pharmacokinetic and pharmacodynamic properties of CDDO-ME are currently being studied for their potency in the treatment of patients with chronic kidney disease, cancer, and pulmonary arterial hypertension [121]. For an effective and safe antiviral therapeutic, the small molecule-driven actin dynamic inhibition strategy must be infected-cell specific. A potential hurdle in the small molecule therapeutic is efficient, targeted and localized delivery. With the size of the nano-scale range (10–100 nm), nanoparticles are an attractive candidate to carry the drug for drug delivery [122]. The ability to conjugate antibodies to the nanoparticles provides the specificity needed for targeted drug delivery. Polyethylene glycol (PEG) based linker has been used for antibody-nanoparticle conjugation [123]. Humanized-antibodies, [124] specific to RSV F protein, can be utilized on the nanoparticle-based strategy to target RSV-infected cells in the respiratory epithelium. For respiratory drug delivery, the nebulization strategy [125] can be employed for delivering actin inhibitors encapsulated with antibody-nanoparticles.

## 7. Prospective

RSV modulates the ARP2/3 complex-driven actin polymerization to induce filopodia for its cell-to-cell spread. It is prudent to determine how the RSV-induced cytoskeletal modulation contributes to disease pathophysiology. By determining the actin cytoskeleton’s role in RSV-driven lung pathophysiology, we may be able to find the mechanism of disparities in the RSV-induced disease pathophysiology in pediatrics, healthy adults and high-risk adults (those with chronic disease). Further research is necessary to identify whether nanoparticle-based ARP2/3 complex inhibitors can be effective RSV therapeutics.

## Figures and Tables

**Table 1:** Role of actin cytoskeleton in RSV replicative cycle.

Steps in RSV Replicative Cycle	Involvement of Actin Cytoskeleton	References
Cell entry	Actin contributes to macropinocytosis and dendritic cell entry.	[35,70,71]
Transcription	Both G-actin and F-actin contribute to initiating RSV transcription. Profilin involves in actin-dependent RSV transcription.	[37,65]
Replication	In vitro studies confirmed that actin, but not microtubulin, is involved in virus replication.	[37,39]
Assembly and budding	F-actin and ARP2 contribute to virus assembly and budding, e.g., the ARP2/3 complex driven viral RNP complex migration.	[37,39,41,66,72]
Cell-to-cell spread	ARP2 and virus-induced filopodia contribute to RSV cell-to-cell spread.	[41,73,74]

## Data Availability

Not applicable.
